# Undergarment Needs and Challenges for Breast Cancer Survivors: A Qualitative Study

**DOI:** 10.21203/rs.3.rs-4307935/v1

**Published:** 2024-05-09

**Authors:** Yen-Tung Liu, Novera H. Khan, Krista M. Nicklaus, Marie Karen Bravo Moix, Chi Liu, Gregory P. Reece, Ashleigh M. Francis, Margaret J. Roubaud, Mia K. Markey

**Affiliations:** The University of Texas at Austin; The University of Texas at Austin; The University of Texas at Austin; Apparel and Art Design College, Xi’an Polytechnic University; Apparel and Art Design College, Xi’an Polytechnic University; The University of Texas MD Anderson Cancer Center; The University of Texas MD Anderson Cancer Center; The University of Texas MD Anderson Cancer Center; The University of Texas at Austin

**Keywords:** Breast reconstruction, Bra experience, Breast cancer, Quality of life, Qualitative study

## Abstract

**Purpose:**

Breast cancer surgery, even with reconstruction, can make it difficult for patients to find a bra that fits properly, is comfortable, and meets their aesthetic standards. We explored breast cancer survivors’ experiences with bras over time to identify preferences, needs, and challenges throughout their journeys.

**Methods:**

Fifteen women who had undergone mastectomy and either delayed or immediate breast reconstruction participated in the study. Focus groups were conducted to explore the participants’ current experiences with bras. They were also prompted to recall their experiences before mastectomy and immediately after reconstruction. The discussion included bra materials, styles, construction techniques, color, quality, and price.

**Results:**

Thematic analysis generated five major themes: “Sense of normalcy and personal well-being,” “Struggles immediately following surgery,” “Transitions in bra experiences and preference,” “Practicality with outfit,” and “Association between quality and price”.

**Conclusion:**

Breast cancer survivors’ well-being is linked to their experiences with bras and the associated purchasing process, and bra needs change throughout the cancer care journey. Survivors’ experiences with bras impact their sense of normalcy and sense of control over significant bodily changes arising from cancer and its treatment. The study underscores the importance of future research on examining the relationship between survivors’ quality of life and garment experiences, including factors such as color choices, closure options, and adjustability for individual needs.

## Introduction

Breast cancer and its treatment often pose challenges concerning the fit, comfort, and aesthetic considerations of undergarments, even for patients who undergo reconstructive procedures, which can impact survivors’ quality of life [1–5]. Previous research used questionnaires [1–3] and interviews [4, 5] to study the bra needs and preferences of breast cancer survivors. LaBat’s survey [1] revealed that nearly one-third of breast reconstruction survivors stated that they no longer wear bras, citing reasons such as the belief that a bra is unnecessary, uncomfortable, or nonconforming to the shape of the reconstructed breast. Likewise, our survey found that ready-to-wear bras are inadequate (e.g., poor fit, difficult to don/doff) and that patients’ received insufficient guidance on bra selection [2]. Wroblewski’s survey [3] demonstrated that a single bra is unlikely to meet the diverse needs and preferences of breast cancer survivors. Survivors who have undergone mastectomy and breast reconstruction expressed heightened concern with underarm and underband comfort [3]. Dhawan’s interviews discovered low bra satisfaction rates among survivors due to perceived comfort and quality issues [5]. Furthermore Jackson’s interviews found that comfort issues arose rapidly as breast cancer survivors adjusted their wardrobes and dressing patterns to accommodate healing and changes in their bodies [4].

However, prior work does not elucidate how garment preferences change over a patient’s breast cancer journey, Understanding preferences at each time point is important for providing guidance aligned with a patient’s current needs and to help patients manage their expectations. This focus group study sought to explore breast cancer survivors’ experiences with bras. In addition to describing their current bra experiences, study participants were prompted to recall their bra experiences before mastectomy and immediately after reconstruction. The goal was to identify the ways in which their experiences with bras changed or remained the same throughout their breast cancer journeys.

## Methods

### Participants and procedures

Prospective study participants completed a set of screening questions in a Qualtrics (Provo, Utah) survey distributed in recruitment materials (e.g., flyers). Eligible participants self-reported that they had previously undergone mastectomy and reconstruction for breast cancer, were at least 6 months past their initial reconstruction surgery, and wore bras on a regular basis at the time of the study. Participants provided informed consent under institutional review board protocol #2016–04-0045 approved by The University of Texas at Austin.

Participants were assigned letters in place of their names (e.g., A, B, C) to ensure confidentiality and facilitate data analysis. Demographic information, medical history, and psychosocial measures were collected using Qualtrics prior to the focus group discussion. In-person focus groups were co-led by a fashion designer (MKBM), professor in apparel and design (CL), and graduate student (KMN). The discussion considered bra materials, styles, construction techniques, color, quality, and price. Participants were prompted to reflect on their experiences with bras at three time points: before mastectomy, immediately after reconstruction, and currently. Seven sample bras, selected to represent a wide range of materials, styles, and construction techniques, were made available during the discussion. Participants were asked to place green stickers on areas of bra samples that they deemed favorable; yellow stickers for areas that were a minor concern but not a significant issue; and red stickers for areas they identified as problematic.

### Body image investment

The Appearance Schemas Inventory-Revised (ASI-R) [9] is a 20-item self-report measure assessing body image investment. It includes beliefs and assumptions about the significance and influence of appearance in one’s life. We report the ASI-R composite score, which is calculated by averaging the individual item scores. A higher ASI-R composite score indicates greater body image investment.

### Body image dissatisfaction

The Body Image Scale (BIS) [8] is a 10-item measure of body image dissatisfaction specifically developed for cancer patients experiencing appearance changes. Each item is scored 0 to 3 such that the total score ranges from 0 to 30. A total score of 0 indicates no body image concerns, while higher scores represent increasing body image concerns.

### Data analysis

Two focus group discussions were transcribed (NHK), and thematic analysis was carried out using NVivo software (Lumivero, Denver, Colorado) (YL, NHK). The study employed Braun and Clarke’s six-step reflexive thematic analysis for the analytical process [10]. Throughout the coding process, an intimate apparel designer (MKBM) provided guidance on the definition of bra-related terminology and explanations as needed. Additionally, insights were shared regarding potential participant confusion when describing bra materials and styles from an apparel design perspective. The first author reviewed, refined, and named themes by considering their relation to the data, checked for consistency, and wrote narratives that explained each theme in the context of the research questions.

## Results

### Participants and procedures

Prospective study participants completed a set of screening questions in a Qualtrics (Provo, Utah) survey distributed in recruitment materials (e.g., flyers). Eligible participants self-reported that they had previously undergone mastectomy and reconstruction for breast cancer, were at least 6 months past their initial reconstruction surgery, and wore bras on a regular basis at the time of the study. Participants provided informed consent under institutional review board protocol #2016–04-0045 approved by The University of Texas at Austin.

Participants were assigned letters in place of their names (e.g., A, B, C) to ensure confidentiality and facilitate data analysis. Demographic information, medical history, and psychosocial measures were collected using Qualtrics prior to the focus group discussion. In-person focus groups were co-led by a fashion designer (MKBM), professor in apparel and design (CL), and graduate student (KMN). The discussion considered bra materials, styles, construction techniques, color, quality, and price. Participants were prompted to reflect on their experiences with bras at three time points: before mastectomy, immediately after reconstruction, and currently. Seven sample bras, selected to represent a wide range of materials, styles, and construction techniques, were made available during the discussion. Participants were asked to place green stickers on areas of bra samples that they deemed favorable; yellow stickers for areas that were a minor concern but not a significant issue; and red stickers for areas they identified as problematic.

### Body image investment

Appearance Schemas Inventory-Revised (ASI-R) [9] is a 20-item self-report measure assessing body image investment. It includes beliefs and assumptions about the significance and influence of appearance in one’s life. We report the ASI-R composite score, which is calculated by averaging the individual item scores. A higher ASI-R composite score indicates greater body image investment.

### Body image dissatisfaction

The Body Image Scale (BIS) [8] is a 10-item measure of body image dissatisfaction specifically developed for cancer patients experiencing appearance changes. Each item is scored 0 to 3 such that the total score ranges from 0 to 30. A total score of 0 indicates no body image concerns, while higher scores represent increasing body image concerns.

### Data analysis

Two focus group discussions were transcribed (NHK), and thematic analysis was carried out using NVivo software (Lumivero, Denver, Colorado) (YL, NHK). The study employed Braun and Clarke’s six-step reflexive thematic analysis for the analytical process [10]. Throughout the coding process, an intimate apparel designer (MKBM) provided guidance on the definition of bra-related terminology and explanations as needed. Additionally, insights were shared regarding potential participant confusion when describing bra materials and styles from an apparel design perspective. The first author reviewed, refined, and named themes by considering their relation to the data, checked for consistency, and wrote narratives that explained each theme in the context of the research questions.

## Discussion

Theme 1 explored how bra color, price, style, and construction technique contributed to personal well-being and a sense of normalcy. Participants reported a strong emotional connection to colors as they symbolize a return to normalcy and happiness. Bright and cheerful colors evoke happiness and energy, while plain and dark colors convey seriousness or depression [13]. Breast cancer survivors frequently feel a loss of control when dealing with a cancer diagnosis and the ability to make garment choices (e.g., colors) can restore some sense of control. Breast cancer survivors have undergone significant challenges and seek items that can contribute to making life feel more comfortable and enjoyable. However, many breast cancer survivors do not have the financial privilege to prioritize happiness and comfort over cost. Similarly, we observed a tradeoff between enhancing a sense of normalcy and comfort. For example, some participants were willing to wear molded cups when they felt that molded cups enhanced their sense of normalcy, even if the cups were not properly fitted. Differing views on padding and molded cups also highlight the need for customization to cater to breast cancer survivors’ unique needs due to substantial variation in the bodily changes resulting from cancer treatment.

Themes 2 and 3 illustrate bra needs change throughout the breast cancer journey, beyond changes that are typical for other bra wearers over their lifespans [14]. In the immediate post-surgery phase, nearly all participants wore front-closure surgical bras due to their restricted movement. However, some participants were frustrated with the hook-and-loop closures, citing discomfort from rubbing on sensitive skin, and with the perceived high price ($100) for a disliked product. Complaints about uncomfortable hook-and-loop closures and front closures seem to be two different issues that are hard to differentiate in this study. Moving beyond the active healing phase, nearly all participants chose to return to back-closure bras, with the most dominant reason being its habitual use. Such habits seem to help patients feel more secure during challenging times. People often use fashion as emotional armor, choosing specific styles for a sense of security. Emotional attachment to clothing can profoundly impact mood by providing familiarity and solace [13, 15]. Another reason participants returned to back-closure bras is the limited adjustability of front closures. Zipper or slider formats are confined to a single size with no flexibility for adjustment. Adjustability is important for accommodating typical fluctuations in body shape and sensitivity. Hook-and-loop or hook-and-eye front closures offer adjustability similar to back-closure bras, but adjusting them can compromise cup shape. Moreover, hook-and-loop closures can cause irritation if not positioned correctly. Future work exploring breast cancer survivors’ perceptions of the textures of hook-and-loop closures could help inform bra design for this population.

Theme 3 also highlights challenges and frustrations participants face in finding bras that meet their specific needs and preferences. Despite receiving suggestions from healthcare providers, and even with the assistance of a bra fitter, the search for a suitable bra can extend for months without success. General suggestions, such as avoiding underwire, do not expedite the search process as this is only one facet of appropriate bra fit. Having assistance from a professional garment fitter can guide both the selection and fitting process, potentially shorten the search time. However, these services are limited to large medical centers and specialty retail stores [1]. Additionally, there are extra charges for the services of a professional mastectomy fitter, and the fitters in retail stores may not have specialized training on the needs of breast cancer patients [16]. Breast cancer survivors report many challenges and frustrations with bras due to an absence or scarcity of suitable options available in the market. For example, participants expressed interest in front closure bras, but reported a lack of comfortable and adjustable front-closure bra options on the market.

## Conclusion

The personal well-being of breast cancer survivors is associated with their experiences wearing bras and the bra purchasing process. Their bra needs change throughout the cancer care journey, and they require more support and guidance during this process. The findings of this study highlight the need for future work to further explore the relationship between breast cancer survivors’ quality of life and the garment experience, with particular emphasis on availability of choices (e.g., colors), closure options (e.g., hook-and-loop vs. hook-and-eye), and adjustability/customization for individual needs (e.g., atypical breast shape).

## Figures and Tables

**Figure 1:**
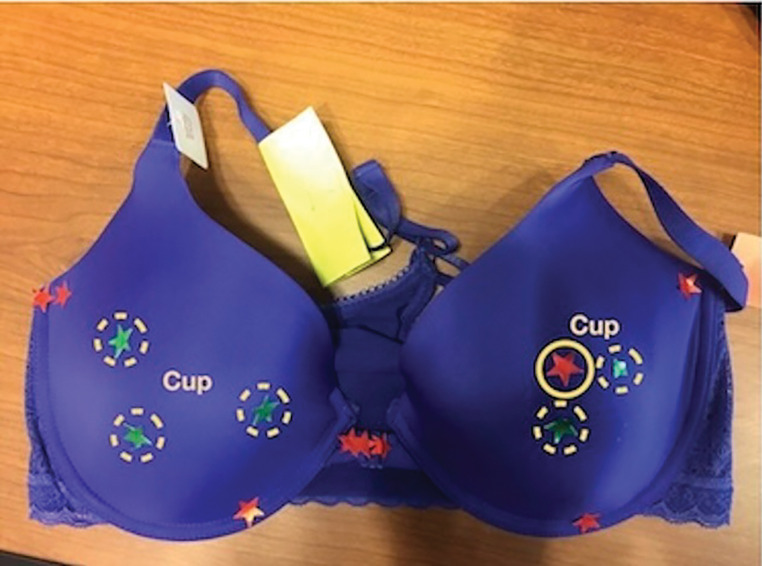
Participants expressed varied sentiments regarding the cup area. They appreciated the concept of molded cups for concealing the abnormal breast shape resulting from surgery, yet they were frustrated by the gap between the breast and the cup, as it failed to accommodate the atypical shape effectively and forced reconstructed breasts to adhere to the molded shape. (Focus group II) Green stickers (dashed line) deemed favorable; red (solid line) identified as problematic.

**Figure 2:**
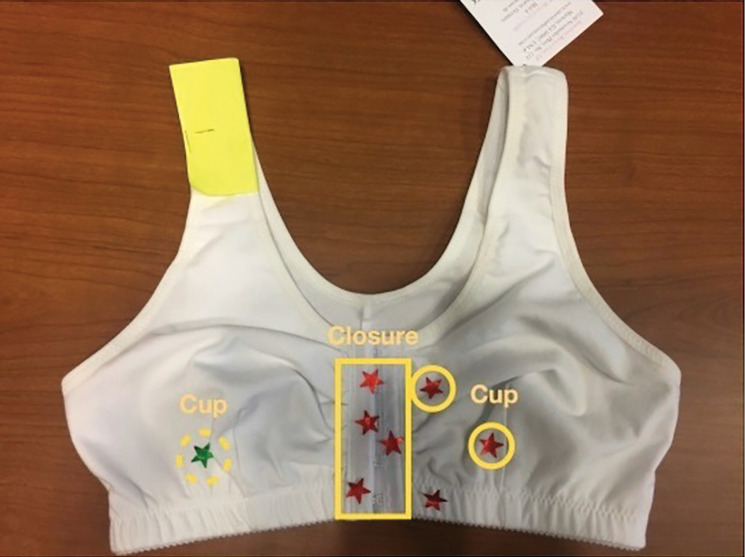
The closure area was marked with numerous red stickers, signaling participants’ discontent with the front hook-and-eye closure. Despite healthcare providers recommending front closure surgical bras for post-surgery recovery, many participants expressed a dislike for this feature. Several participants specifically identified this bra as the one they wore immediately after surgery. Additionally, there were mixed feelings about the dart-shaped cup, which is designed to create a shape to better accommodate the breast. (Focus group II) Green stickers (dashed line) deemed favorable; red (solid line) identified as problematic.

**Figure 3:**
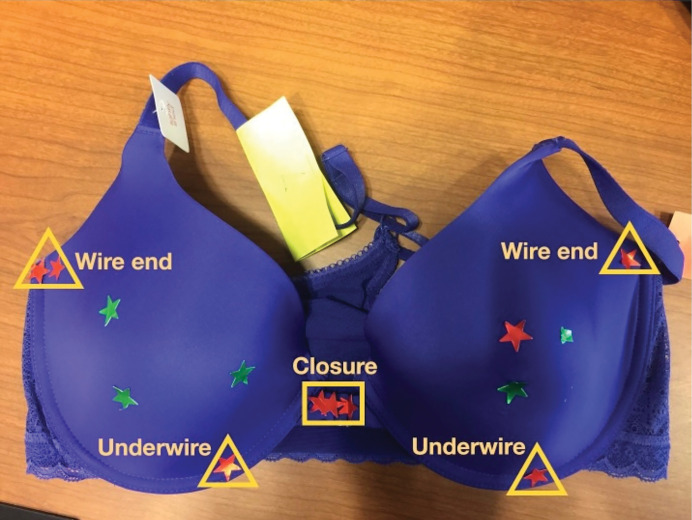
Participants expressed their dislike of underwire bras, which aligned with their dissatisfaction with front-closure bras. The underwire section was marked with red indicators, as participants found the underwires unbearable, particularly below the breast where their incisions (i.e., Wise patterns [11, 12]) were located. The end part of the underwire was also marked due to its excessive hardness on the skin. (Focus group II) Green stickers (dashed line) deemed favorable; red (solid line) identified as problematic.

**Table 1.** Sample characteristics and medical variables (n = 15). The demographic profi les of participants aligns withthe characteristics typically observed in the breast cancer population in the US [6]. Likewise, the mean ASI-Rcomposite score for this study is comparable to ASI-R composite values previously reported for the breastreconstruction population, e.g., [7]. BIS had good clinical validity in our sample, with only one item failing toreach the 30% response rate criterion (avoiding people because of feelings about physical appearance) [8].Participants in this study endorsed more body image concerns (i.e., higher mean BIS) than participants inthe study that established the scale [8]. One possible explanation is that the inclusion criteria for breastcancer survivors in this study required a post-surgery timeframe of only 6 months, as opposed to thevalidation study of the instrument in which the minimum post-surgery time was 1 year.

Characteristic		n	%
Age	(Mean, SD, range) = (50.67, 10.14, 30–64)		
Ethnicity	Hispanic or Latino	2	13.33
	Not Hispanic or Latino	13	86.67
Relationship status	Married/Domestic partnership	9	60
	Committed relationship	2	13.33
	Single	2	13.33
	Divorced	2	13.33
Employment status	Employed or self-employed	13	86.67
	Retired	1	6.67
	Stay-at-home caregiver	1	6.67
Total household income	$26,000–$50,000	2	13.33
	$51,000–$75,000	3	20
	$76,000–$100,000	5	33.33
	$101,000–$150,000	4	26.67
	Greater than $150,000	1	6.67
Highest degree earned	High school diploma or GED	1	6.67
	Associate’s degree	2	13.33
	Bachelor’s degree	6	40
	Master’s degree	4	26.67
	Doctoral degree	2	13.33
Laterality of reconstruction	Unilateral	4	26.67
	Bilateral	11	73.33
Timing of reconstruction	Immediate	10	66.67
	Delayed	5	33.33
Type of reconstruction	Autologous	10	66.67
	Implant	4	26.67
	Combination	1	6.67
BMI	(Mean, SD, range) = (29.89, 7.67, 20.30–46.80)		
ASI-R	(Mean, SD, range) = (2.99, 0.30, 2.20–3.35)		
BIS	(Mean, SD, range) = (11.8, 6.24, 1–24)		

Abbreviations: BMI, body mass index; ASI-R, Appearance Schemas Inventory-Revised; BIS, Body Image Scale

**Table 2.** Participant demographic details

Participant	Age	Ethnicity	Relationship status	Employment status	Total household income	Highest degree earned
E1	51	Not Hispanic or Latino	Married/Domestic Partnership	Employed or self-employed	$76,000–$100,000	Bachelors degree
D1	53	Not Hispanic or Latino	Married/Domestic Partnership	Employed or self-employed	$76,000–$100,000	Bachelors degree
A	35	Hispanic or Latino	Married/Domestic Partnership	Employed or self-employed	$26,000–$50,000	Associate degree
B	55	Not Hispanic or Latino	Single	Employed or self-employed	$51,000–$75,000	Doctoral degree
C	56	Not Hispanic or Latino	Married/Domestic Partnership	Employed or self-employed	$76,000–$100,000	Bachelors degree
D	47	Not Hispanic or Latino	Married/Domestic Partnership	Employed or self-employed	$101,000–$150,000	Bachelors degree
E	61	Not Hispanic or Latino	Married/Domestic Partnership	Retired	Greater than $150,000	Bachelors degree
F	64	Not Hispanic or Latino	Married/Domestic Partnership	Employed or self-employed	$101,000–$150,000	Masters degree
G	38	Not Hispanic or Latino	Married/Domestic Partnership	Employed or self-employed	$101,000–$150,000	Masters degree
H	56	Hispanic or Latino	Committed Relationship	Employed or self-employed	$51,000–$75,000	Associate degree
I	30	Not Hispanic or Latino	Committed Relationship	Employed or self-employed	$51,000–$75,000	Masters degree
J	44	Not Hispanic or Latino	Single	Employed or self-employed	$76,000–$100,000	Doctoral degree
K	62	Not Hispanic or Latino	Divorced	Employed or self-employed	$76,000–$100,000	Masters degree
L	50	Not Hispanic or Latino	Married/Domestic Partnership	Stay-at-home caregivers	$101,000–$150,000	Bachelors degree
M	58	Not Hispanic or Latino	Divorced	Employed or self-employed	$26,000–$50,000	High school diploma or GED

**Table 3.** Medical details by participant

Participant	BMI	ASI-R	BIS	Laterality	Reconstruction timing	Reconstruction type
E1	34.5	2.85	15	Bilateral	Immediate	Autologous
D1	25.8	3.25	15	Bilateral	Delayed	Autologous
A	30.7	2.9	2	Bilateral	Immediate	Autologous
B	39.6	3.25	15	Unilateral	Immediate	Autologous
C	23.3	2.7	6	Unilateral	Immediate	Autologous
D	25	2.75	12	Bilateral	Delayed	Implant
E	22.5	3.05	16	Unilateral	Immediate	Implant
F	38.5	3.3	24	Bilateral	Immediate	Implant
G	22.9	3.15	9	Bilateral	Delayed	Implant
H	22.7	3.1	14	Bilateral	Delayed	Autologous
I	31	2.2	12	Bilateral	Immediate	Autologous
J	33.6	3.05	18	Bilateral	Immediate	Autologous
K	46.8	3.35	14	Unilateral	Delayed	Autologous
L	20.3	2.9	1	Bilateral	Immediate	Hybrid
M	31.1	3.1	5	Bilateral	Immediate	Autologous

Abbreviations: BMI, body mass index; ASI-R, Appearance Schemas Inventory-Revised; BIS, Body Image Scale
